# Assessment and comparison of rhizosphere communities in cultivated *Vaccinium* spp. provide a baseline for study of causative agents in decline

**DOI:** 10.3389/fpls.2023.1173023

**Published:** 2023-06-27

**Authors:** Joseph Kawash, Peter V. Oudemans, Lindsay Erndwein, James J. Polashock

**Affiliations:** ^1^USDA-ARS, Genetic Improvement of Fruits and Vegetables Laboratory, Chatsworth, NJ, United States; ^2^Department of Plant Biology, Rutgers University, New Brunswick, NJ, United States; ^3^Oak Ridge Institute for Science and Education (ORISE) Postdoctoral Scholar, USDA-ARS, Genetic Improvement of Fruits and Vegetables Laboratory, Chatsworth, NJ, United States

**Keywords:** *Vaccinium corymbosum*, *Vaccinium macrocarpon*, phytobiome, acidic soil, soil health

## Abstract

It has long been recognized that the community of organisms associated with plant roots is a critical component of the phytobiome and can directly or indirectly contribute to the overall health of the plant. The rhizosphere microbial community is influenced by a number of factors including the soil type, the species of plants growing in those soils, and in the case of cultivated plants, the management practices associated with crop production. *Vaccinium* species, such as highbush blueberry and American cranberry, are woody perennials that grow in sandy, acidic soils with low to moderate levels of organic matter and a paucity of nutrients. When properly maintained, fields planted with these crops remain productive for many years. In some cases, however, yields and fruit quality decline over time, and it is suspected that degenerating soil health and/or changes in the rhizosphere microbiome are contributing factors. Determining the assemblage of bacterial and fungal microorganisms typically associated with the rhizosphere of these crops is a critical first step toward addressing the complex issue of soil health. We hypothesized that since blueberry and cranberry are in the same genus and grow in similar soils, that their associated rhizosphere microbial communities would be similar to each other. We analyzed the eukaryotic (primarily fungal) and bacterial communities from the rhizosphere of representative blueberry and cranberry plants growing in commercial fields in New Jersey. The data presented herein show that while the bacterial communities between the crops is very similar, the fungal communities associated with each crop are quite different. These results provide a framework for examining microbial components that might contribute to the health of *Vaccinium* spp. crops in New Jersey and other parts of the northeastern U.S.

## Introduction

Highbush Blueberry (*Vaccinium corymbosum* L.) and large-fruited or American cranberry (*V. macrocarpon* Ait.) are long-lived woody perennials that are native to North America. Cultivars of both species are clonally propagated. Blueberry is propagated from hardwood or softwood cuttings or *in vitro* culture and rooted prior to field planting (Mainland, 1966). Cranberry cuttings can be pressed directly into field soil or rooted prior to transplanting into the field (Eck, 1990). Establishment time can vary depending on planting location, but in the northeastern U.S., it typically takes blueberry fields 4-7 years to reach maturity. New plantings of cranberry similarly take about 4-5 years to reach full production. Once established, plantings may remain productive for many years. However, the yield of mature plantings of blueberry and cranberry sometimes declines over time. In areas where decline is prevalent, and replanting does not solve the problem, the primary cause is likely to be soil-related.

It is well established that long-term monoculture can reduce soil health (Ketcheson, 1980; Porter et al., 1997; Garside et al., 2001; Bennett et al., 2012). This decline of soil health can be complex and includes, but is not limited to, a buildup of salts and/or other toxic materials, reduction in organic matter, and a buildup of detrimental soil organisms (Tewoldemedhin et al., 2011; Mazzola and Manici, 2012). Poor performance of replanted blueberry fields, termed Blueberry replant disease (BRD) in the southeastern U.S., was found to be primarily due to pathogenic nematodes (Jagdale et al., 2013), while cranberry decline in Wisconsin was associated with *Phytophthora* spp. (Jeffers, 1988). In contrast, blueberry and cranberry decline in New Jersey, does not seem to be associated with either *Phytophthora* spp. or nematodes, although in some replanted blueberry fields where experimental nematicides were tested, plant growth improved (Oudemans, unpublished). Thus, it is likely that the cause(s) of decline in either crop varies by locality and may be due to complex community interactions, rather than a single pathogenic organism.

Blueberry and cranberry naturally grow in well-drained, sandy acidic (pH 4.0-5.5) soils that tend to be low in nutrients and with low to moderate (2-7 percent) organic matter (Eck and Childers, 1966; Eck, 1988; Eck, 1990, Pavlis et al., unpublished). Since the soils in which these crops grow is not typical, as compared to the soils in which most crops are grown, the assemblage of microorganisms in these soils is likely to be unique. While we expect to find a range of acidophilic organisms, Fierer (2017) suggested that abiotic stressors, including acidic conditions, can limit microbial survival and growth. He further notes that soil bacterial communities and abundances of specific taxa can be predicted from site characteristics which include soil pH (Fierer, 2017).

In addition to soil type, the plants growing in the soil have a profound effect on the rhizosphere microbiome (Costa et al., 2006; Berendsen et al., 2012; Marschner, 2012; Chaparro et al., 2014). We expected that because highbush blueberry and cranberry are in the same genus (*Vaccinium*) and grow naturally in similar soils and environments, that there would be significant commonalities in the rhizosphere communities associated with these crops. However, plant habit (1-2 m high shrub for blueberry; low-growing 0.2 -0.6 m high vines for cranberry) and management practices differ between these crops and these differences might drive some distinctions in the rhizosphere communities of these crops.

Management practices differ greatly between blueberry and cranberry. Highbush blueberry in the northeastern U.S. is still grown primarily in clean culture, i.e. all competing vegetation is controlled by tilling and herbicide use (Ballinger, 1966). The plants are pruned to remove old wood and to encourage the growth of 2-3 year old productive shoots. The plants are deciduous and go dormant for the winter. In contrast, cranberry is grown in solid stands of vines in beds of varying size with little or no exposed soil. Weeds are pulled by hand or controlled with herbicides to form a near-monoculture of cranberry vines. Cranberry plants retain most of their leaves at the end of the growing season, but still go dormant for the winter.

In this study, we sought to determine and compare the rhizosphere microbiome (bacteria and fungi) of cultivated cranberry and blueberry fields in New Jersey as a prerequisite to exploring the cause(s) of decline. While we predicted commonalities among the microbial communities based on similarity of soil type and plant genus, management practices and plant growth habit were expected to cause a divergence in at least some aspects of the microbial communities.

## Materials and methods

### Plant material and collection

Blueberry rhizosphere soil samples were collected from 16 mature (over 10 years old) fields (2 samples/field) across 4 different commercial farms (3 in Atlantic County, NJ, USA and 1 in Burlington County, NJ, USA), for a total of 32 samples. An additional paired blueberry soil sample was collected from a 10-year old research field at the P.E. Marucci Blueberry and Cranberry Research Station (Burlington County, NJ, USA) (**Table S1**). Rhizosphere soil was collected, with a small shovel, from the root zone (between 30 and 40 cm from the crown) of 3-4 mature blueberry plants per sample location to a depth of 16 cm and homogenized in buckets. The homogenized soil in each bucket was considered to be one sample. Cranberry soils from mature beds (over 10 years old) were collected from 8 fields (4 samples/field) from a commercial farm (E, **Table S1**) in Burlington County, NJ, USA (32 samples). One extra paired sample was collected from commercial farm F (**Table S1**). The 34 cranberry soil samples were collected with a soil probe to a depth of 10 cm. The core, containing the roots of 3-4 samples per field were homogenized and the roots removed before DNA isolation. All blueberry and cranberry samples were collected on 22 or 23 July, 2015.

### DNA extraction and amplification

Total DNA was extracted from the soil samples using the PowerLyzer PowerSoil DNA Isolation kit (MoBio Laboratories, Carlsbad, CA, USA), but with protocol modifications suggested by the manufacturer for soils with low biomass and based on our own experience. Briefly, 0.5 g of each soil sample was added to the dry glass bead tubes, followed by 500 µl Bead Solution, 200 µl of phenol:chloroform (1:1) pH 8.0, and 60 µl of C1 solution. Samples were processed in a TissueLyser II (Qiagen, Germantown, MD, USA) at a frequency of 20 Hz for 2 x 5 min and the slurry was transferred to Phase Lock Gel Light 2 ml tubes (5 Prime, Hilden, Germany). The tubes were centrifuged at 10,000 x g for 1 min and the supernatants were transferred to clean 2 ml tubes. The remainder of the protocol was as per the manufacturer’s standard protocol.

The DNA samples were processed for sequencing by Molecular Research LP (Shallowater, TX, USA). Diagnostic portions of the DNA were amplified using PCR. Specifically, the 16S rRNA gene V4 variable region primers 515/806 (Caporaso et al., 2011) and the rDNA ITS region primers ITS1/4 (White et al., 1990) were used to assay bacteria and fungi respectively.

### DNA sequencing and computational processing

All amplified DNA fragments were sequenced using the Illumina MiSeq (Illumina, San Diego, CA, USA) platform. Operational taxonomic unit (OTU) picking was performed by Molecular Research LP (Shallowater, TX, USA) using an in-house pipeline developed by the provider (Molecular Research) from protocols described in; (Dowd et al., 2008a; Dowd et al., 2008b; Edgar, 2010; Capone et al., 2011; Eren et al., 2011; Swanson et al., 2011). Reads were separated by sample barcodes, followed by removal of sequences not meeting quality criteria, including short sequences < 200bp, sequences with ambiguous base calls, and sequences with homopolymer runs exceeding 6bp. Singletons and chimeras were also removed. The reads were then separated into bins and the binned sequences were clustered into OTUs at 97% similarity. Final OTUs were taxonomically classified from a database derived from GreenGenes (DeSantis et al., 2006), RDPII, (http://rdp.cme.msu.edu), and NCBI (www.ncbi.nlm.nih.gov) using BLASTn. 16S and 18S OTU tables for lowbush blueberry in Nova Scotia, Canada were kindly provided by S. Yurgel (Yurgel et al., 2017). 18S OTU tables for cultivated grape were provided by M. Chou (Chou et al., 2018).

### Statistical analysis and graph generation

Alpha diversity was calculated using the QIIME analysis toolkit (Caporaso et al., 2010), specifically, the alpha_diversity.py script using the Shannon index. OTU tables for bacteria and fungi were processed individually. Analysis of variance (ANOVA) was used to compare alpha diversity between blueberry and cranberry for each sample group.

Differential abundance and statistical significance (assigned at 0.05) of OTUs between blueberry and cranberry were calculated using the Analysis of Compositions of Microbiomes (ANCOM) R package utilizing the provided stringent correction for multiple testing (Mandal et al., 2015). OTU tables for the bacteria and fungi were analyzed independently. Subsequent boxplots of differentially abundant OTUs were generated using a python script with NumPy and Matplotlib modules (Van der Walt et al., 2011).

Assignment of guilds for fungi was performed using a python script to query the FUNGuild database (Nguyen et al., 2016). OTU tables were OTU tables were parsed using python, specifying a fungal database search. All guild assignments were filtered as being plant-relevant unless otherwise noted.

Diversity graphs were generated with a python script utilizing the NetworkX package (Hagberg et al., 2008) and Matplotlib (Hunter, 2007) modules for creating the display. Each entry on the taxonomic tree starting with an individual phylum present in the collective OTU set is represented by a node, with nodes linked through descent of the taxonomic hierarchy to the family rank. Hence, following a given phylum are all of the classes that are associated with that phylum present in the OTU table. Unique OTUs found within a given taxon were counted and attributed to node area. Therefore, nodes with increased diversity will be larger, having several connecting nodes with a variety of lower taxa associated, or with few connecting edges, indicating a large number of less diverse taxa. Shading of the node ranged from blue (r,g,b:0,0,1) for blueberry and red (r,g,b:1,0,0) for cranberry. The corresponding node color was therefore a ratio of the number of OTUs found in cranberry versus blueberry (cranberry_OTUs/total_OTUs,0, blueberry_OTUs/total_OTUs). Taxa that are more diverse in cranberry samples are shaded increasingly red, while taxa more diverse in blueberry samples are shaded blue. The arrangement of nodes for display was established by the NetworkX fruchterman_reingold _layout method.

Circular graphs of the percent abundance of genera were generated using GraPhiAn (Asnicar et al., 2015). The imputed taxonomic trees were generated using the hierarchy of the OTU assignments from each OTU table (bacterial or fungal). The datasets were filtered to display only OTUs from the respective kingdom that were classifiable to the genus level. Corresponding graph values for each OTU represent the percent (as a number of total reads assigned to that particular OTU, out of all assigned reads) abundance of a given taxonomic level. Abundance equal to or exceeding 0.5% was annotated for graphical representation of contributing taxa.

## Results

### Fungi

**Figure 1:**
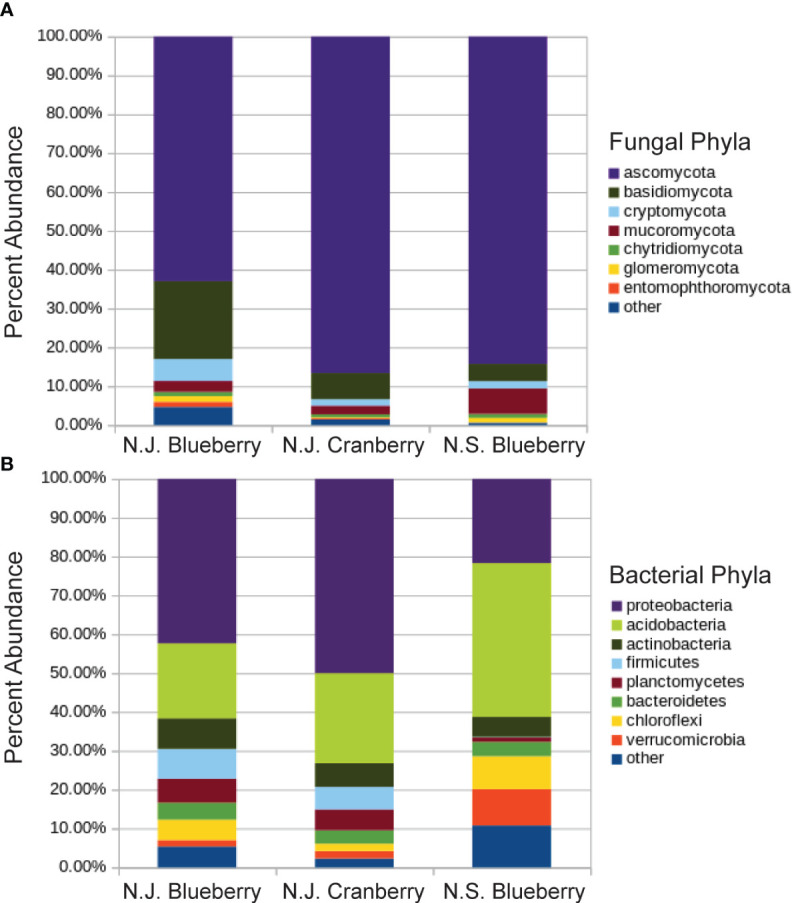
Percent abundance of fungal **(A)** and bacterial **(B)** phyla in the rhizosphere of cultivated highbush blueberry and cranberry in New Jersey (N.J.). Data for lowbush blueberry from Nova Scotia (Yurgel et al., 2017) shown for comparison.

The fungal analysis resulted in 1.26M reads matched to OTUs for blueberry and 1.35M reads for cranberry, averaging 38.2K reads and 39.7K reads per sample respectively. Both groups were dominated by the phyla Ascomycota, 63.0% and 88.6%, and Basidiomycota, 20.0% and 6.9%, in blueberry and cranberry respectively (**Figure 1A**). The percent abundance of major fungal phyla varied between blueberry and cranberry, and included; Chytridiomycota, Mucoromycota, Cryptomycota, Etomophthoromycota, and Glomeromycota (**Figure 1A**). However, no significant differential abundance between blueberry and cranberry was identified by ANCOM for any of these phyla.

**Figure 2:**
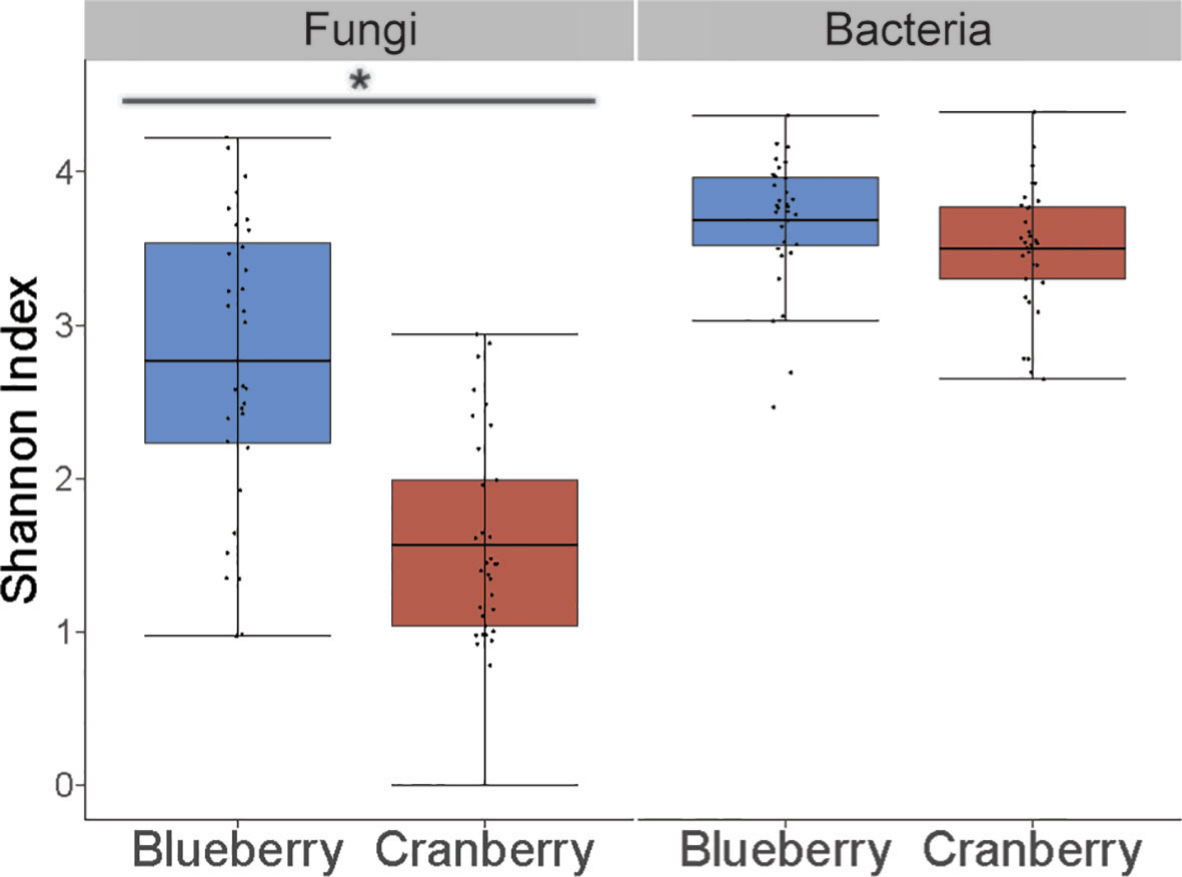
Alpha diversity (Shannon index) in the rhizosphere of cultivated highbush blueberry and cranberry in New Jersey (N.J.) for fungi and bacteria sampling. Horizontal line within each box represents the mean. Whiskers extend to the last data points that fall within 1.5 times the interquartile range from either the first or third quartile. Asterisk indicates significant difference between samples (*P* < 0.001).

**Figure 3:**
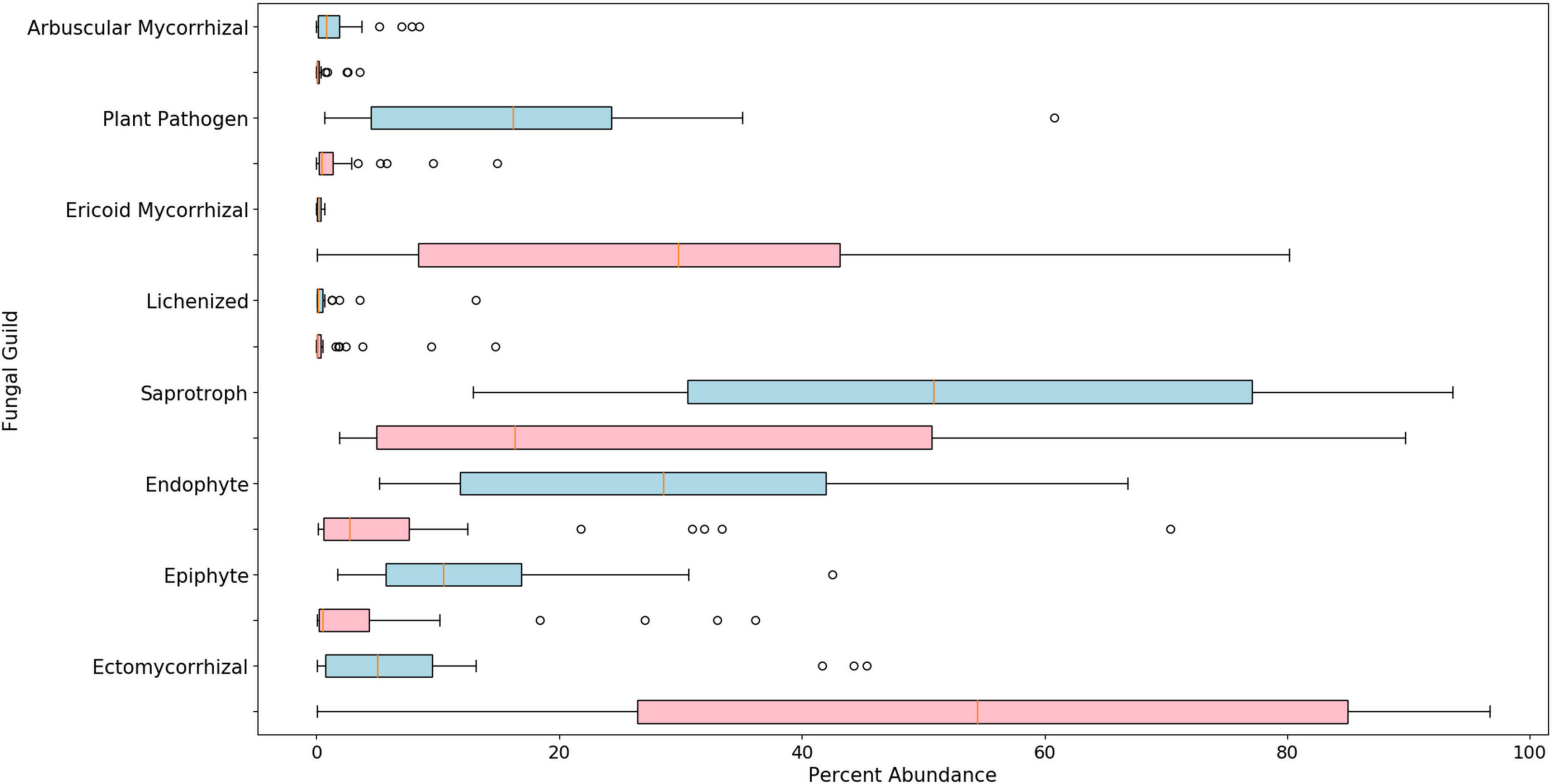
Boxplots of relative abundance for classified fungi in highbush blueberry (blue) and cranberry (red) in New Jersey. The vertical line within each box represents the median value. The shaded area extends from the first through the third quartiles. Whiskers extend to the last data points that fall within 1.5 times the interquartile range from either the first or third quartile. All data beyond these bounds are deemed outliers and are represented by individual circles.

There were 753 fungal genera identified using these methods, but the relative abundance of the majority (722) was below 1% (**Figures S1**, **S2**). The alpha diversity, based on the Shannon Index, was found to be significantly different between blueberry and cranberry (**Figure 2**). Most of the fungi that were classified using the FUNGuild database, at the genus level, with an abundance of 0.5% or greater, were saprotrophs (22) followed by symbionts (16), plant pathogens (8), endophytes (7), and epiphytes (3) (**Figure 3**).

**Figure 4:**
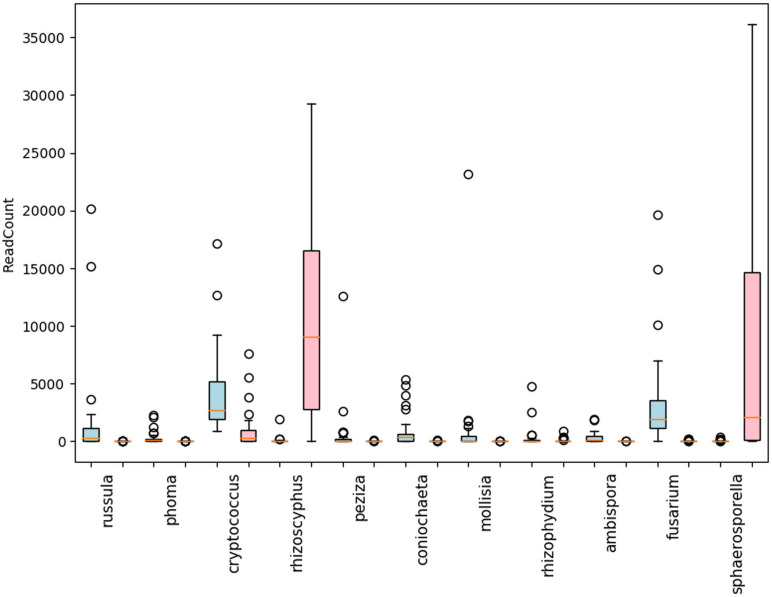
Differentially abundant fungal genera with mean read counts greater than 200 in the rhizosphere of cultivated highbush blueberry (blue) and cranberry (red) in New Jersey, according to ANCOM. The horizontal line within each box represents the median value, the shaded area extends from the first through the third quartiles. Whiskers extend to the last points that fall within 1.5 times the interquartile range from either the first or third quartile. All data beyond these bounds are deemed outliers and are represented by individual circles.

Within the shared (blueberry and cranberry) rhizosphere fungi, significant differential abundance was found in the pathogenic genera *Fusarium* (7.0% and 0.0%), *Phoma* (1.0% and 0.0%), *Rhizophydium* (0.5% and 0.7%), *Coniochaeta (3.6% and 0.0)*, and *Mollisia* (3.2% and 0.0%) (**Figure 4**). Blueberry also contained several fungal pathogens nearly uniquely (extremely low evidence, below 0.1% in cranberry) in its rhizosphere microbiome, though each represented less than 1% of the genus content. These pathogens included *Athelia* (0.7%), *Alternaria* (0.4%) and *Cladosporium* (0.3%).

Of the symbionts in the blueberry and cranberry soils (16 genera were 0.5% or greater), 4 (*Phialophora* 0.1% and 0.5%, *Rhizoscyphus* 0.2% and 32.4%*, Oidiodendron* 0.0% and 0.1%, and *Hymenoscyphus* 0.0% and 0.5%*)* are known ericoid mycorrhizae. Nine other genera of mycorrhizal (ecto and arbuscular) fungi were also detected at abundances of 0.5% or greater, that are not generally thought to be associated with ericoid plants. Differentially abundant non-ericoid mycorrhizal fungi included (blueberry and cranberry), *Ambispora* (1.2% and 0.0%), *Peziza* (2.1% and 0.0%), *Russula* (5.3% and 0.1%), *Sphaerosporella* (0.1% and 24.8%), (**Figure 4**). *Sarcosphaera* (0.4% and 0.1%) was found most abundant in blueberry soil, but not significantly so. Endophyte and epiphyte classified genera were also detected with significant differential abundance including *Cryptococcus* (13.8% and 5.6%), and *Mollisia* (3.2% and 0.0%) (**Figure 4**).

**Figure 5:**
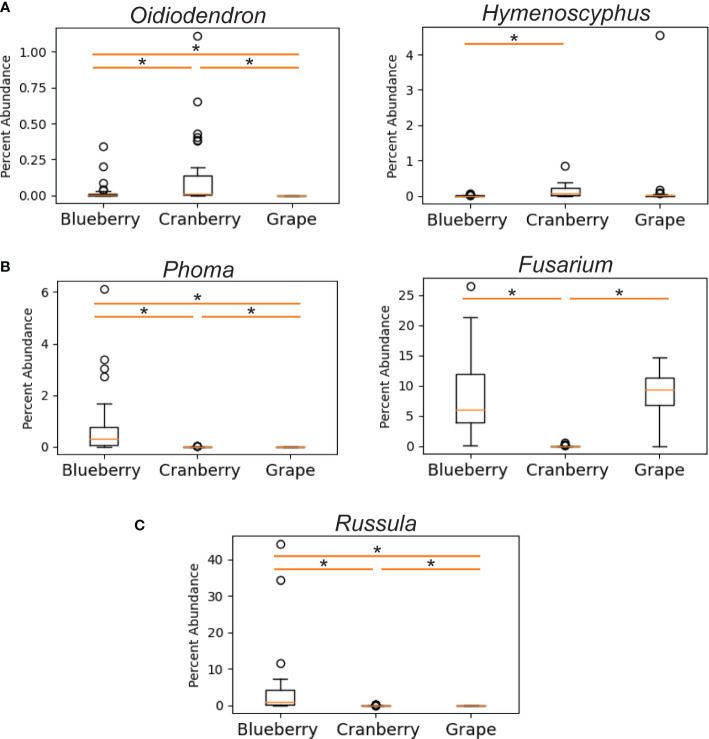
Differentially abundant fungal genera as a percentage of reads identified in the rhizosphere of cultivated highbush blueberry (left) and cranberry (center) in New Jersey, and cultivated grape (right) in New York (Chou et al., 2018). Fungal genera are categorized by: ericoid mycorrhizae **(A)**, pathogens **(B)**, and ectomycorrhizae **(C)**. The horizontal line within each box represents the median value, the boxed area extends from the first through the third quartiles. Whiskers extend to the last points that fall within 1.5 times the interquartile range from either the first or third quartile. All data beyond these bounds are deemed outliers and are represented by individual circles. Asterisk indicates significant difference between samples (*P* < 0.05).

We also compared differential abundance between both blueberry and cranberry soils with grape, taking note in particular of significant pathogenic and symbiotic fungi from cranberry and blueberry soils. Profiles of grape symbiotic fungal abundance were also relatively low and did not trend towards either blueberry or cranberry. The grape profile was similar to cranberry, for *Russula* and similar to blueberry, for *Oidiodendron* and *Hymenoscyphus* (**Figure 5A**). Of the pathogenic fungi, grape shared a similar *Fusarium* profile with blueberry, having a significantly high abundance compared to cranberry. However, the grape profile for the pathogenic fungus *Phoma*, had little similarity with blueberry, and greater resemblance to cranberry, although all three groups were significantly different from one another (**Figure 5B**). *Rusulla*, a genus containing ectomycorrhizal mushrooms was found to significantly differ across all three crops (**Figure 5C**).

### Bacteria

In the bacterial analysis, 2.38M reads were matched with OTUs in blueberry, averaging 72.1K reads per sample, and 2.31M reads in cranberry, averaging 67.9K reads per sample. There was little difference in the makeup of the bacterial microbiome at the phylum level for blueberry vs. cranberry (**Figure 1B**). The order of dominance, however, differed between the two (blueberry vs. cranberry), though Proteobacteria (42.4% and 50.0%) and Acidobacteria (19.3% and 23.1%) were the most prevalent for both N.J. crops. High numbers of bacteria, from 5 other phyla [Planctomyces (6.2% and 5.4%), Verrucomicobia (1.6% and 1.9%), Bacteriodetes (4.4% and 3.5%), Firmicutes (7.6% and 5.8%), and Chloroflexi (5.2% and 1.9%)] were also detected. Any unique phyla found in either group represented less than 1% abundance in the rhizosphere microbiome. Even at the genus level, unique taxa were largely identified at an abundance below 1% (**Figures S3**, **S4**). Alpha diversity was not significantly different between blueberry and cranberry for bacteria OTUs (**Figure 2**).

Though no known bacterial pathogens were identified at the genus level as being significantly differentially present between blueberry and cranberry at any reasonable abundance, *Pseudomonas* (0.2% and 0.1%), *Burkholderia* (0.4% and 0.3%), *Candidatus* (genus 2.9% and 1.9%), *Saccharimonas* (0.2% and 0.3%), and *Rhizobium* (2.1% and 1.3%) were all found in the rhizosphere microbiome.

## Discussion

The fungi were remarkably similar at the phylum level, as there were no notable omissions between the rhizosphere microbiome of blueberry or cranberry. They only differed in the hierarchical abundance within phyla groups (**Figure 1A**), and not significantly so. Although blueberry had a significantly higher alpha diversity compared to cranberry, potentially due to distinguishing agricultural practices and possibly plant growth habit. Cranberry is typically cultivated in wetlands and this may explain why the chytridiomycota, a phylum common in aquatic environments (Barr, 1990; Gleason et al., 2008), is prevalent in the cranberry soils. In fact, the chytrid *Synchytrium vaccinii* is a known pathogen of cranberry and requires free water to spread (Stretch and Carris, 2017). However, fungi from this phylum were not exclusive to cranberry as they were also found in both the New Jersey (N.J.) and the Nova Scotia (N.S.) blueberry soils (**Figure 1A**). These findings parallel other reports of *Vaccinium* spp. soils, especially the identification of ascomycota and basidiomycota as major composition groups, though not with absolute ranking similarities (Li et al., 2020; Morvan et al., 2020; Zhou et al., 2022).

Many fungal genera were classified that have not been previously recognized as inhabiting the soils associated with *Vaccinium* spp. in the northeastern U.S. (**Figures S1**, **S2**). Detection of unique genera is not surprising considering; 1) that these methods are more sensitive than traditional isolation and culturing methods and 2) attention is typically directed toward organisms that are pathogenic or beneficial to the crop in question, thus, neutral organisms and those found in low abundance are often not characterized and/or reported. We classified the fungal genera into guilds where possible, including pathotrophs (plant, fungal, and animal), saprotrophs, symbionts, epiphytes, and endophytes. While most of the detected organisms were saprotrophs, as would be expected to colonize organic soil matter, several plant pathogens were also identified at abundances of 0.5% or greater. The presence of pathogens in the soil, even at low levels, may signal a potential disease problem since these pathogens may serve as a reservoir for an outbreak that could express under disease-conducive conditions.

**Figure 6:**
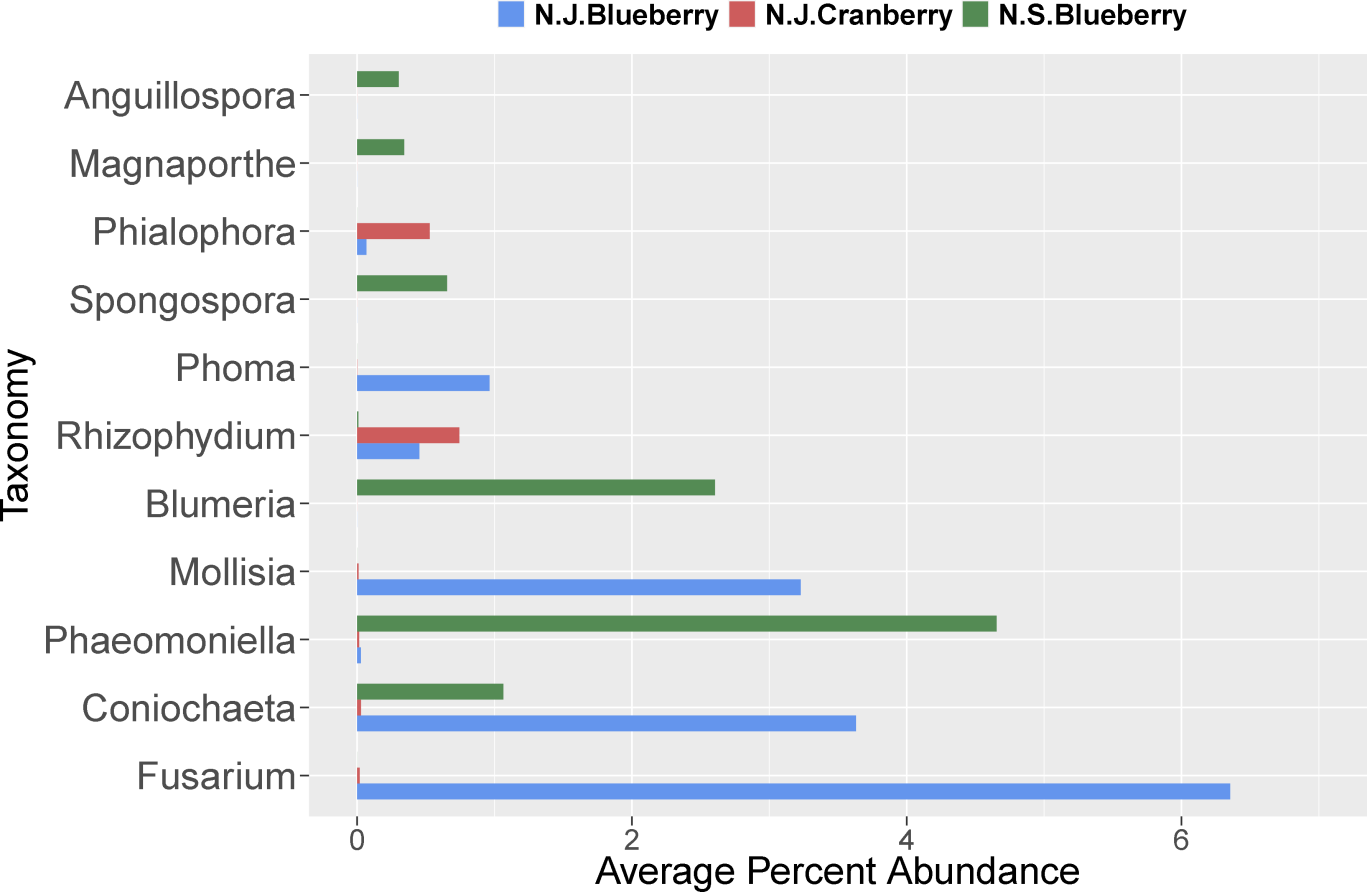
The average percent abundance of the identified plant pathogenic fungal genera in the rhizosphere of cultivated highbush blueberry (blue) and cranberry (red) in New Jersey (N.J.). Data for lowbush blueberry (green) from Nova Scotia (Yurgel et al., 2017) shown for comparison.

Generally, of the classifiable fungal genera, blueberry contained a higher pathogen load. For example, *Coniochaeta* spp. are known to be pathogens of woody perennials and thus is a potential pathogen of these crops (Damm et al., 2010). *Athelia rolfsii* causes Southern blight of solanaceous crops, beans and many other plants, but has not been reported as a pathogen of *Vaccinium* spp. (Aycock, 1961). *Alternaria* spp. and *Cladosporium* spp. are known to be pathogens of blueberry and cranberry (Polashock et al., 2017). Overall, N.J. blueberry contained a higher percent abundance of the identifiable potential pathogens *Coniochaeta*, *Fusarium*, *Mollisia*, and *Phoma* compared to cranberry, with several samples harboring statistical outliers of pathogen abundance far above average (**Figure 4**, represented as circles). In addition, *Blumeria*, a species of which causes powdery mildew on grasses, was uniquely identified in N.S. blueberries (**Figure 6**). N.J. cranberry however, had a comparatively low pathogen load, and low pathogen diversity (**Figures 3**, **4**, **6**).

Several symbionts were also identified in the rhizosphere microbiome of N.J. blueberry and N.J. cranberry, including ericoid, ecto, and arbuscular mycorrhizae. These may represent important, but previously unknown, *Vaccinium* spp.- associated mycorrhizae. N.J. cranberry had a particularly high average abundance of *Rhizoscyphus* (note that *Rhizoscyphus ericae* = *Hymenoscyphus ericae*, but our data generally are not able to reach the species level and both genera are still listed in the databases used- hence both are used in this paper), compared to N.J. blueberry samples (**Figure 3**). Interestingly, it has been reported that mycorrhizae that proliferate in response to crop monoculture, may in fact be detrimental to that crop (Johnson et al., 1992). While endophytes and epiphytes were also detected, their importance in blueberry and cranberry production is not currently known. Similar to southern highbush blueberry, neither N.J. blueberry or N.J. cranberry carried a particularly high load of *Oidiodendron*, though cranberry carried significantly more than blueberry (Li et al., 2020 and **Figure 5**).

We also compared known symbionts and pathogens with another small fruit crop grown in the northeast, grape. Similar to blueberry, grape exhibited very low levels of both *Oidiodendron* sp. and *Hymenoscyphus* sp., both of which are known to be ericoid mycorhizzae (**Figure 5A**), though no evidence of *Russula* sp. was found (**Figure 5C**). The analysis of pathogens showed that grape and blueberry show a similarly high abundance of *Fusarium* sp. (**Figure 5B**), which is shown to be a potential cause of decline in grape (Highet and Nair, 1995; Bustamante et al., 2022).

When compared to findings in southern highbush, as described in Li et al. (2020), Hyaloscyphaceae, Leotiaceae, Pezoloma, and Hyaloscypha were not found in meaningful abundance in N.J. blueberry and N.J. cranberry.

Bacterial composition was similar between N.J. blueberry, N.J. cranberry, and N.S. blueberry. One of the top phyla however, chloroflexi, was found to differ significantly in abundance between blueberry and cranberry soils. Similarity in composition continued at the genus level, where little difference was found between N.J. blueberry and N.J. cranberry at abundances greater than 1.0%. Both N.J. blueberry and N.J. cranberry were also found to have similar bacterial alpha diversity indexes. As compared to southern highbush blueberry (Li et al., 2020), both N.J. blueberry and N.J. cranberry contained high levels of proteobacteria, acidobacteria, and actinobacteria making up the majority of bacterial phyla. This trend followed other studies that identified similarly high abundance of proteobacteria, acidobacteria, and actinobacteria in *Vaccinium* soils (Li et al., 2020; Morvan et al., 2020; Zhou et al., 2022). However, both N.J. blueberry and N.J. cranberry contained a heavier load of Firmicutes than southern highbush blueberry.

Only a small number of diseases of blueberry and cranberry are known to be caused by bacteria, with some of the agents being; *Pseudomonas syringae*, *Xylella fastidiosa*, *Burkholderia andropogonis*, *Agrobacterium* spp., *Rhizobium* spp. and *Candidatus* spp. (which cause phytoplasma diseases) (Polashock et al., 2017). At the genus level, several of these pathogens were identified at similar abundance in N.J. blueberry and N.J. cranberry rhizospheres including *Pseudomonas, Burkholderia, Rhizobium, and Candidatus*, although generally at low average abundances (<0.5%) (**Figures S3**, **S4**).

**Figure 7:**
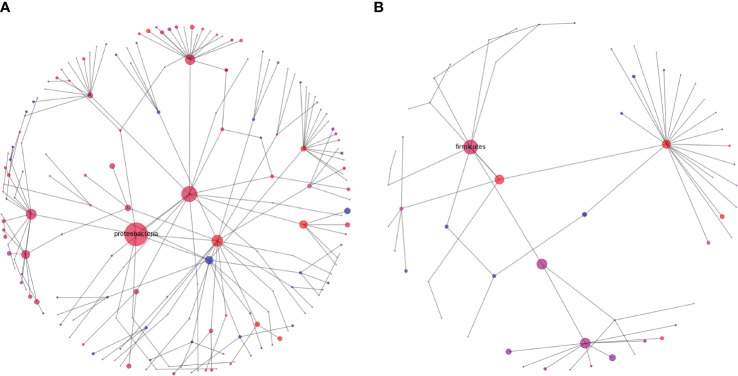
Diversity graphs of unique OTUs found in the bacteria, Proteobacteria **(A)** and Firmicutes **(B)**, from the rhizosphere soil of highbush blueberry and cranberry from New Jersey. Head nodes are labeled by phylum, lower nodes follow taxonomic hierarchy identified in available OTUs to the family level. Node area is proportional to the number of unique OTUs identified. Node color is a proportion of the number of unique highbush blueberry (blue) OTUs as compared to unique cranberry (red) OTUs.

**Figure 8:**
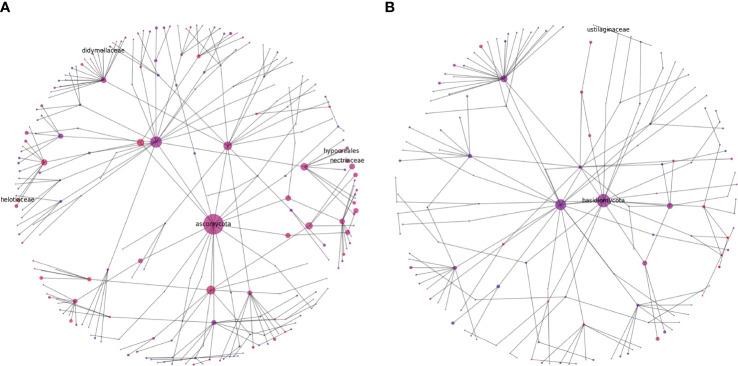
Diversity graphs of unique OTUs found in fungi, Ascomycota **(A)** and Basidiomycota **(B)**, from the rhizosphere soil of highbush blueberry and cranberry from New Jersey. Head nodes are labeled by phylum, lower nodes follow taxonomic hierarchy identified in available OTUs to the family level. Node area is proportional to the number of unique OTUs identified. Node color is a proportion of the number of unique highbush blueberry (blue) OTUs as compared to unique cranberry (red) OTUs. Notable pathogen and symbiont families mentioned in the text are also labeled.

Rhizophere microbiome diversity is thought to be an important indicator of soil health (Berendsen et al., 2012; Wallenstein, 2017; Schloter et al., 2018), and comparison in diversity can help to elucidate potential pathogenic influences, nutritional weaknesses, or microbial niches that have developed in differing soil samples. To help in visualizing these complex communities, we generated representations of microbial diversity for the largest and most diverse bacterial and fungal phyla (**Figures 7**, **8**). The Proteobacteria exhibited a high level of diversity, as indicated by the relatively high number of large hubs, of various intermediate colors, and a high level of connectivity between nodes. There do remain, however, a few nodes that appear to be dominated in either the cranberry or blueberry groups, suggesting that niches have developed for particular taxa, or that the ecosystem does not support the other associated organisms. Conversely in Firmicutes, there are few noteworthy classes that are attached to the parent phylum. Though the phylum Firmicutes may not be as large as that of Proteobacteria, of the two largest classes represented here, Firmicutes appears to be more dominated by the cranberry group. This potentially indicates that there is a lack of diversity of Firmicutes in blueberry.

Sequencing methods and bioinformatics tools are evolving rapidly and application of this technology allows us to gain insight into the rhizosphere microbiome, a key component of the phytobiome that impacts plant health. The OTUs generated from these samples were used to identify primarily fungi and bacteria that comprise the microbial communities that are associated with the rhizosphere of blueberries and cranberries in New Jersey. While we found similar communities between blueberry and cranberry in New Jersey, we noted differences between our data and that of the lowbush blueberry rhizosphere in Nova Scotia. Although lowbush blueberry (*Vaccinium angustifolium*) is in the same genus, the environmental conditions, management practices and soil characteristics are very different between the two locations (N.J. and Nova Scotia, Canada). Where similarity may still occur, is in characterizing the factors associated with poor plant performance as many of the diseases that are known to affect these crops overlap (Polashock et al., 2017). Using these methods, allows the detection and subsequent targeting of specific organisms using chemical control and/or modification of cultural practices. At the same time, enhancement of beneficial organisms, such as ericoid mycorrhizae, can also be monitored.

Many years ago, Waksman (Waksman, 1918) stated “The cranberry soils are so distinctly different from ordinary soils that it was thought for a long time that no very large number of bacteria can exist in them and that the microbial population consists predominantly of molds”. Though the uniqueness of the soils in which cranberries (and blueberries) holds true, we have shown that there are many phyla of bacteria and fungi that are adapted to these crops and the environments in which they grow. As we further characterize this myriad of organisms, we ultimately hope to characterize the microorganism(s) specifically associated with crop decline.

## Data availability statement

The data presented in the study are deposited in the NCBI Sequence Read Archive under BioProject ID: PRJNA956268

## Author contributions

The project was conceived and directed by JP and PO. PO directed field collections and JP directed all laboratory work. JK and LE performed various data analyses and created the figures. JP and JK wrote the manuscript draft with contributions and editing by PO and LE. All authors contributed to the article and approved the submitted version.

## Supplementary material

Supplementary Figure 1 Diversity of fungal genera from the rhizosphere soil of highbush blueberry from New Jersey. Genera with an average relative abundance greater than 0.5% across samples are labeled. The height of the bars of the outer ring correspond to the average relative abundance. Each color represents a different phylum, and phyla with an average abundance greater than 0.5% are labeled. Tree nodes follow taxonomic hierarchy from kingdom (central) to genus (exterior).

Supplementary Figure 2 Diversity of fungal genera from the rhizosphere soil of cranberry from New Jersey. Genera with an average relative abundance greater than 0.5% across samples are labeled. The heights of the bars of the outer ring correspond to the average relative abundance. Each color represents a different phylum and phyla with an average abundance greater than 0.5% are labeled. Tree nodes follow taxonomic hierarchy from kingdom (central) to genus (exterior).

Supplementary Figure 3 Diversity of bacterial genera from the rhizosphere soil of highbush blueberry from New Jersey. Genera with an average relative abundance greater than 0.5% across samples are labeled. The height of the bars of the outer ring correspond to the average relative abundance. Each color represents a different phylum and phyla with an average abundance greater than 0.5% are labeled. Tree nodes follow taxonomic hierarchy from kingdom (central) to genus (exterior).

Supplementary Figure 4 Diversity of bacterial genera from the rhizosphere soil of cranberry from New Jersey. Genera with an average relative abundance greater than 0.5% across samples are labeled. The heights of the bars of the outer ring correspond to the average relative abundance. Each color represents a different phylum and phyla with an average abundance greater than 0.5% are labeled. Tree nodes follow taxonomic hierarchy from kingdom (central) to genus (exterior).

Supplementary Table 1 Locations, coordinates, and soil types for blueberry and cranberry rhizosphere soil collections.
