# Supplementary figures and images for: Assessment and comparison of rhizosphere communities in cultivated Vaccinium spp. provide a baseline for study of causative agents in decline
Source: Front Plant Sci. 2023 Jun 27;14:1173023. doi: 10.3389/fpls.2023.1173023 (PMC10333580; doi:10.3389/fpls.2023.1173023)

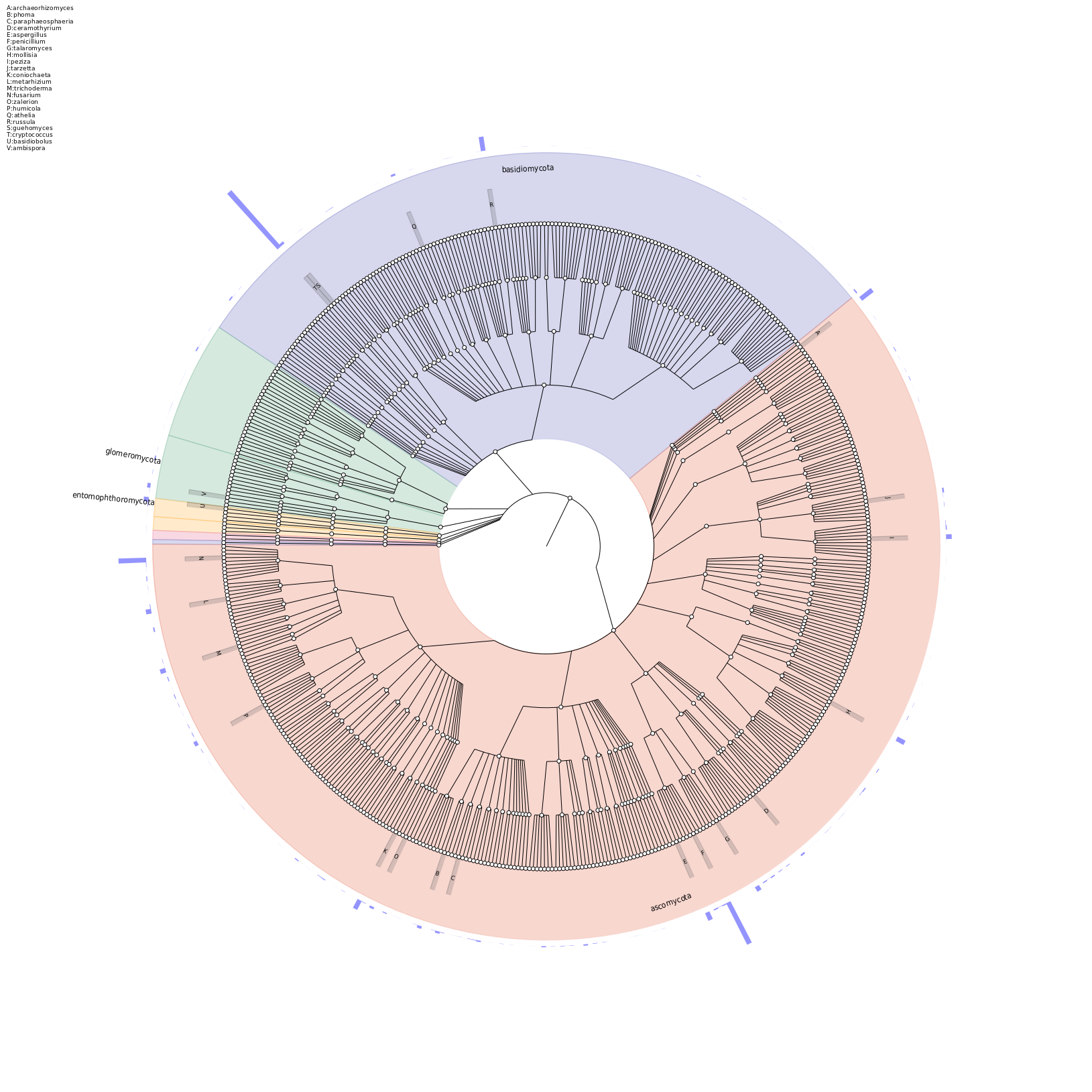

Supplement: Supplementary Figure 1 — Diversity of fungal genera from the rhizosphere soil of highbush blueberry from New Jersey. Genera with an average relative abundance greater than 0.5% across samples are labeled. The height of the bars of the outer ring correspond to the average relative abundance. Each color represents a different phylum, and phyla with an average abundance greater than 0.5% are labeled. Tree nodes follow taxonomic hierarchy from kingdom (central) to genus (exterior). [file Image_1.tiff]

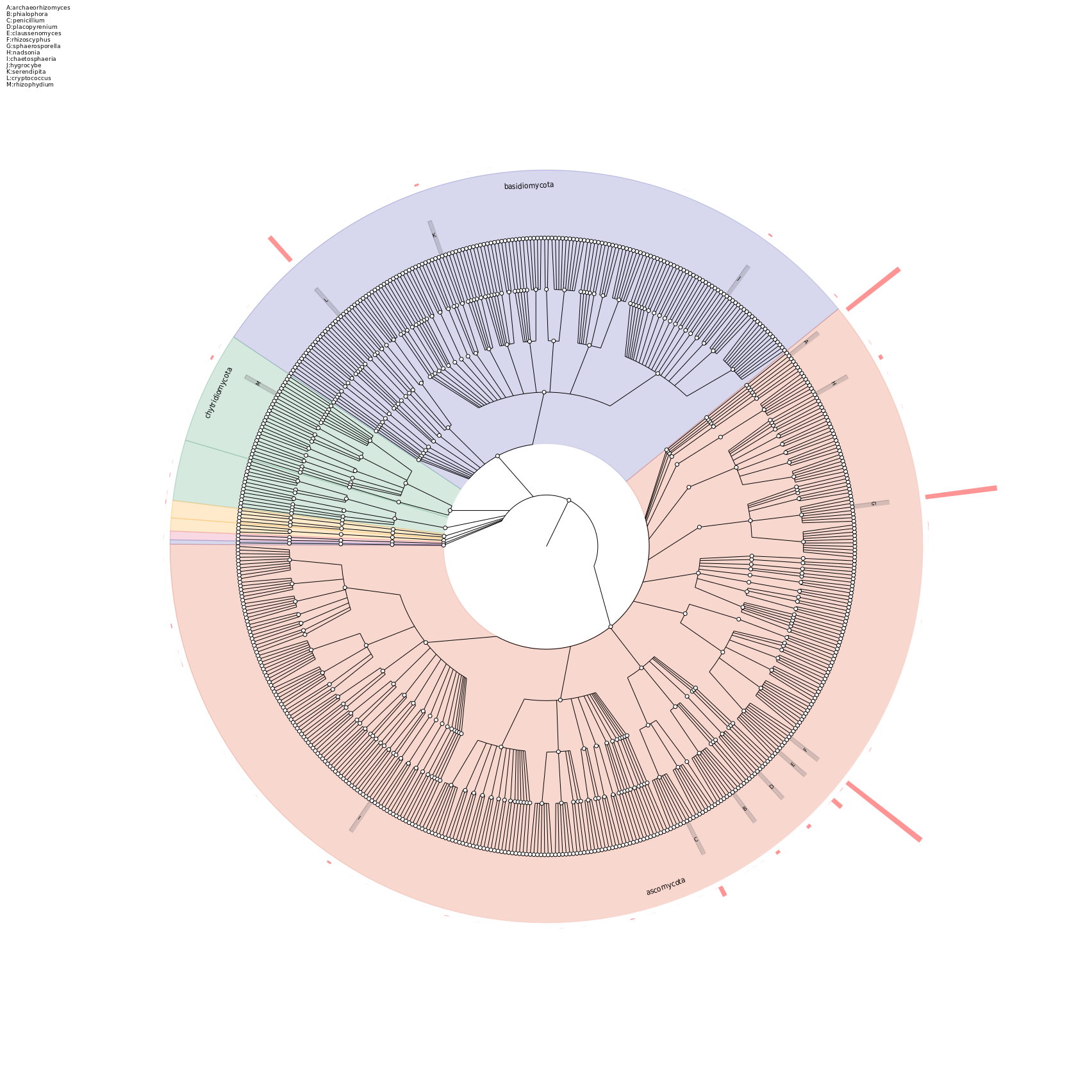

Supplement: Supplementary Figure 2 — Diversity of fungal genera from the rhizosphere soil of cranberry from New Jersey. Genera with an average relative abundance greater than 0.5% across samples are labeled. The heights of the bars of the outer ring correspond to the average relative abundance. Each color represents a different phylum and phyla with an average abundance greater than 0.5% are labeled. Tree nodes follow taxonomic hierarchy from kingdom (central) to genus (exterior). [file Image_2.tiff]

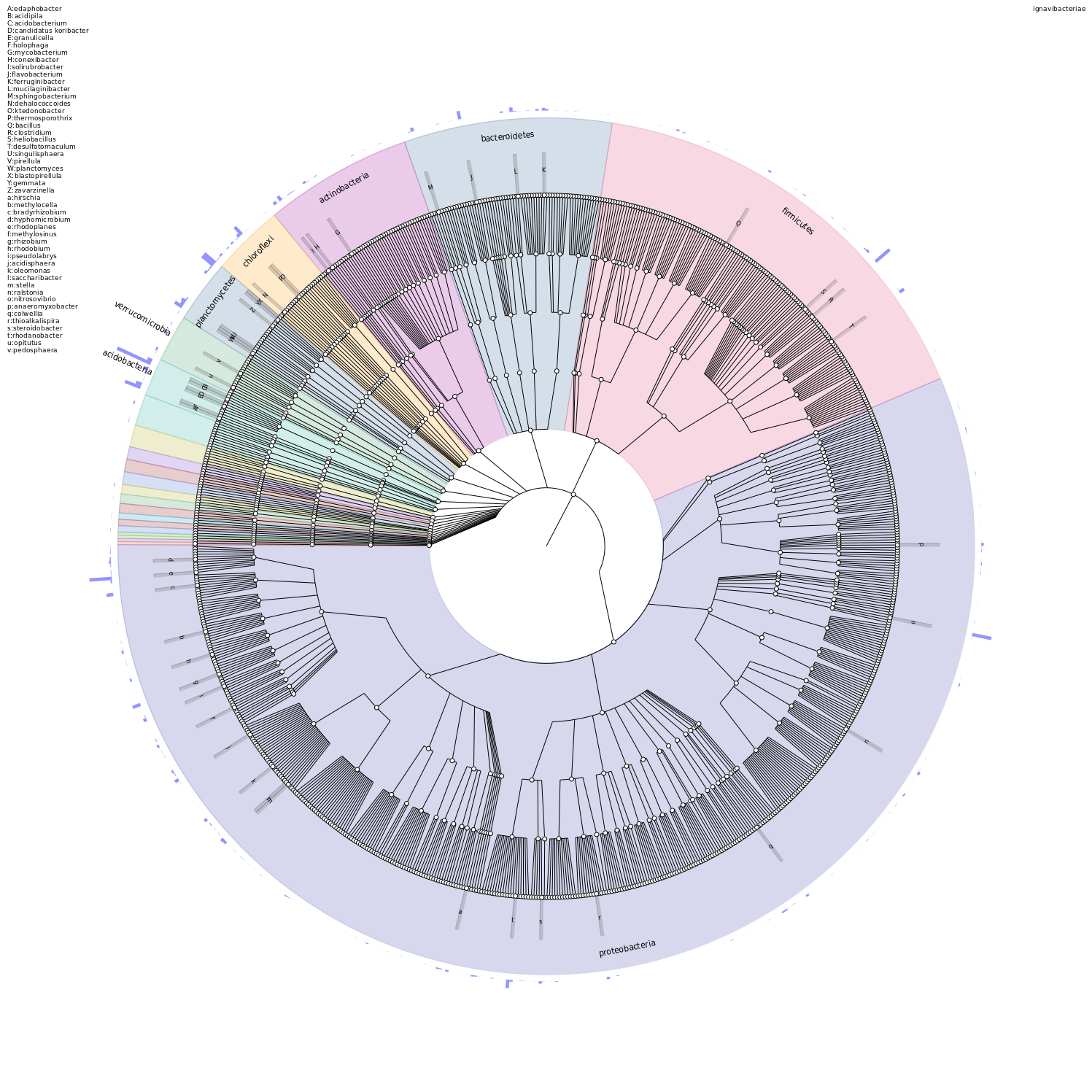

Supplement: Supplementary Figure 3 — Diversity of bacterial genera from the rhizosphere soil of highbush blueberry from New Jersey. Genera with an average relative abundance greater than 0.5% across samples are labeled. The height of the bars of the outer ring correspond to the average relative abundance. Each color represents a different phylum and phyla with an average abundance greater than 0.5% are labeled. Tree nodes follow taxonomic hierarchy from kingdom (central) to genus (exterior). [file Image_3.tiff]

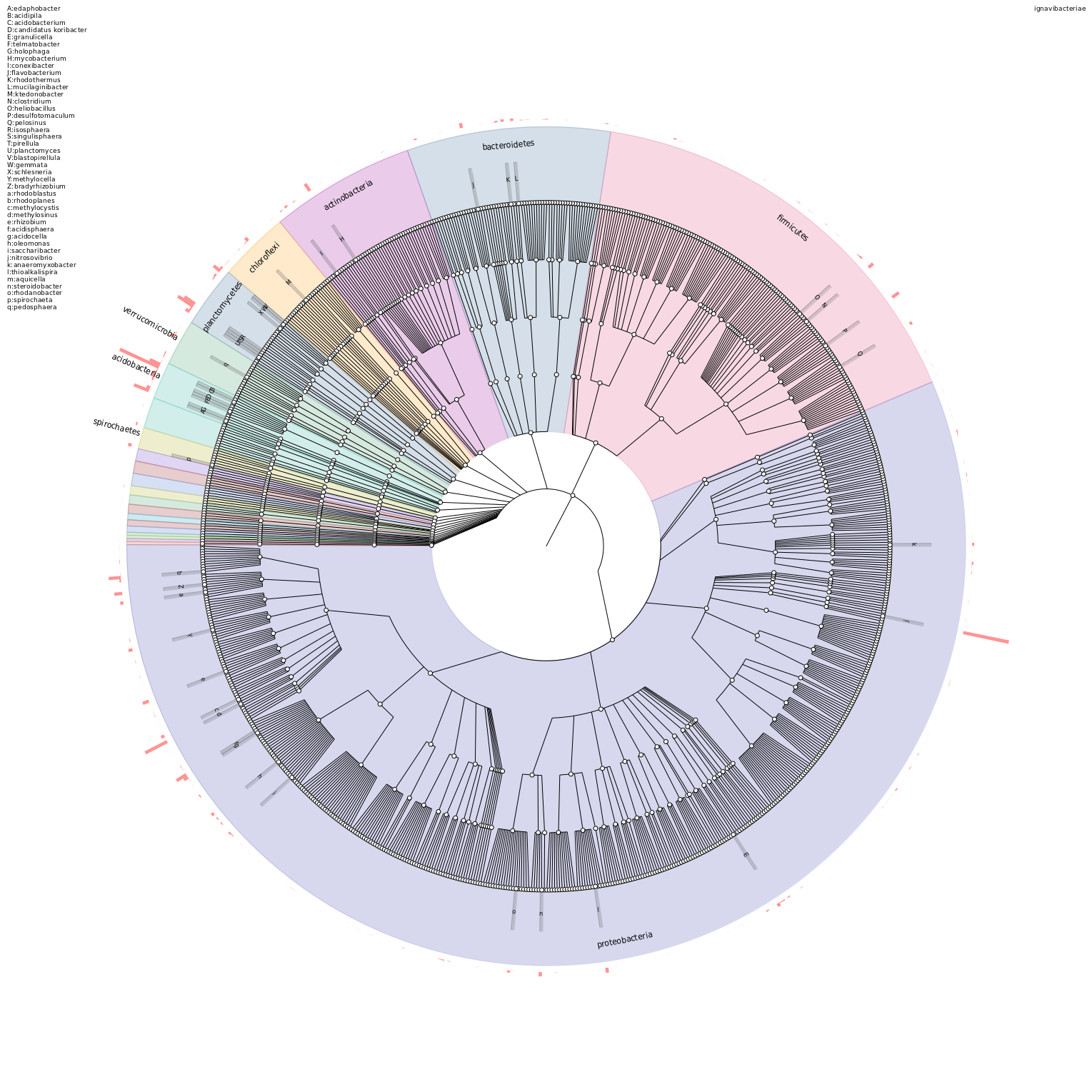

Supplement: Supplementary Figure 4 — Diversity of bacterial genera from the rhizosphere soil of cranberry from New Jersey. Genera with an average relative abundance greater than 0.5% across samples are labeled. The heights of the bars of the outer ring correspond to the average relative abundance. Each color represents a different phylum and phyla with an average abundance greater than 0.5% are labeled. Tree nodes follow taxonomic hierarchy from kingdom (central) to genus (exterior). [file Image_4.tiff]
