# Supplementary material for: Assessment and comparison of rhizosphere communities in cultivated Vaccinium spp. provide a baseline for study of causative agents in decline
Source: Front Plant Sci. 2023 Jun 27;14:1173023. doi: 10.3389/fpls.2023.1173023 (PMC10333580; doi:10.3389/fpls.2023.1173023)
Supplement: Supplementary Table 1 — Locations, coordinates, and soil types for blueberry and cranberry rhizosphere soil collections. [file Table_1.docx]

Table S1.

| **Sample** | **Locale** | **Coordinates** | **County** | **Soil Type** |
| --- | --- | --- | --- | --- |
| **Blueberry** | | | |  |
|  | Farm A | 39°37’45.8”N 74°44’47.3”W | Atlantic | LakB-Lakehurst sand |
|  | Farm B | 39°32’49.7”N 74°46’50.9”W | Atlantic | BerAr-Berryland sand |
|  | Farm C | 39°56’28.1”N 74°37’03.0”W | Burlington | BerAr-Berryland sand |
|  | Farm D | 39°35’39.6”N 74°46’06.1”W | Atlantic | DocBO- Downer loamy sand |
|  | P.E. Marucci Center | 39°43’01.7”N 74°30’20.9”W | Burlington | LakB-Lakehurst sand |
| **Cranberry** | | | |  |
|  | Farm E | 39°44’28.8”N 74°31’40.3”W | Burlington | AtsAO-Atsion sand |
|  | Farm F | 39°57’15.9”N 74°29’55.3”W | Burlington | AtsAO- Atsion sand |
